# Banff Digital Pathology Working Group: Image Bank, Artificial Intelligence Algorithm, and Challenge Trial Developments

**DOI:** 10.3389/ti.2023.11783

**Published:** 2023-10-16

**Authors:** Alton B. Farris, Mariam P. Alexander, Ulysses G. J. Balis, Laura Barisoni, Peter Boor, Roman D. Bülow, Lynn D. Cornell, Anthony J. Demetris, Evan Farkash, Meyke Hermsen, Julien Hogan, Renate Kain, Jesper Kers, Jun Kong, Richard M. Levenson, Alexandre Loupy, Maarten Naesens, Pinaki Sarder, John E. Tomaszewski, Jeroen van der Laak, Dominique van Midden, Yukako Yagi, Kim Solez

**Affiliations:** ^1^ Department of Pathology and Laboratory Medicine, Emory University, Atlanta, GE, United States; ^2^ Department of Laboratory Medicine and Pathology, Mayo Clinic, Rochester, MN, United States; ^3^ Department of Pathology, University of Michigan, Ann Arbor, MI, United States; ^4^ Department of Pathology and Medicine, Duke University, Durham, NC, United States; ^5^ Institute of Pathology, Rheinisch-Westfälische Technische Hochschule (RWTH) Aachen University Clinic, Aachen, Germany; ^6^ Department of Nephrology and Immunology, RWTH Aachen University Clinic, Aachen, Germany; ^7^ Department of Pathology, University of Pittsburgh, Pittsburgh, PA, United States; ^8^ Department of Pathology, Radboud University Medical Center, Nijmegen, Netherlands; ^9^ Nephrology Service, Robert Debré Hospital, University of Paris, Paris, France; ^10^ Department of Pathology, Medical University of Vienna, Vienna, Austria; ^11^ Department of Pathology, Amsterdam University Medical Centers, Amsterdam, Netherlands; ^12^ Department of Pathology, Leiden University Medical Center, Leiden, Netherlands; ^13^ Georgia State University, Atlanta, GA, United States; ^14^ Emory University, Atlanta, GA, United States; ^15^ Department of Pathology, University of California Davis Health System, Sacramento, CA, United States; ^16^ Institut National de la Santé et de la Recherche Médicale, UMR 970, Paris Translational Research Centre for Organ Transplantation, and Kidney Transplant Department, Hôpital Necker, Assistance Publique-Hôpitaux de Paris, University of Paris, Paris, France; ^17^ Department of Microbiology, Immunology and Transplantation, KU Leuven, Leuven, Belgium; ^18^ Division of Nephrology, Hypertension, and Renal Transplantation, Department of Medicine, Intelligent Critical Care Center, College of Medicine, University of Florida at Gainesville, Gainesville, FL, United States; ^19^ Department of Pathology, The State University of New York at Buffalo, Buffalo, NY, United States; ^20^ Center for Medical Image Science and Visualization, Linköping University, Linköping, Sweden; ^21^ Memorial Sloan Kettering Cancer Center, New York, NY, United States; ^22^ Department of Pathology, University of Alberta, Edmonton, AB, Canada

**Keywords:** Banff, digital pathology, artificial intelligence, machine learning, image analysis

## Abstract

The Banff Digital Pathology Working Group (DPWG) was established with the goal to establish a digital pathology repository; develop, validate, and share models for image analysis; and foster collaborations using regular videoconferencing. During the calls, a variety of artificial intelligence (AI)-based support systems for transplantation pathology were presented. Potential collaborations in a competition/trial on AI applied to kidney transplant specimens, including the DIAGGRAFT challenge (staining of biopsies at multiple institutions, pathologists’ visual assessment, and development and validation of new and pre-existing Banff scoring algorithms), were also discussed. To determine the next steps, a survey was conducted, primarily focusing on the feasibility of establishing a digital pathology repository and identifying potential hosts. Sixteen of the 35 respondents (46%) had access to a server hosting a digital pathology repository, with 2 respondents that could serve as a potential host at no cost to the DPWG. The 16 digital pathology repositories collected specimens from various organs, with the largest constituent being kidney (*n* = 12,870 specimens). A DPWG pilot digital pathology repository was established, and there are plans for a competition/trial with the DIAGGRAFT project. Utilizing existing resources and previously established models, the Banff DPWG is establishing new resources for the Banff community.

## Introduction

The Banff Digital Pathology Working Group (DPWG) was formed in 2019, followed by a publication describing the DPWG’s main goals and the current state of transplant digital pathology [1]. Since then, the DPWG meets regularly in video conferences (nearly every 2 weeks) to discuss new digital pathology initiatives, innovative investigations, and digital pathology’s current status and future (2), particularly computer vision applied to transplantation, considering the fact that digital pathology has enabled the development of “computational pathology” as a new science [2–4]. “Computational Pathology” is a novel approach to precision medicine incorporating multiple data sources using artificial intelligence (AI) to generate actionable knowledge to improve disease diagnosis, prognostication, and prediction [5].

The development of new digital pathology-based tools, computer vision algorithms, and machine learning (ML) models for the study of kidney diseases has stimulated the pathology and nephrology community to build large digital pathology repositories to allow for the integration of data from clinical, molecular, pathology, and other domains. While this effort has been in place for over a decade for native kidney diseases [5], the use of digital pathology repositories, computer vision, and computational pathology in transplant pathology remains largely unexplored.

**FIGURE 1:**
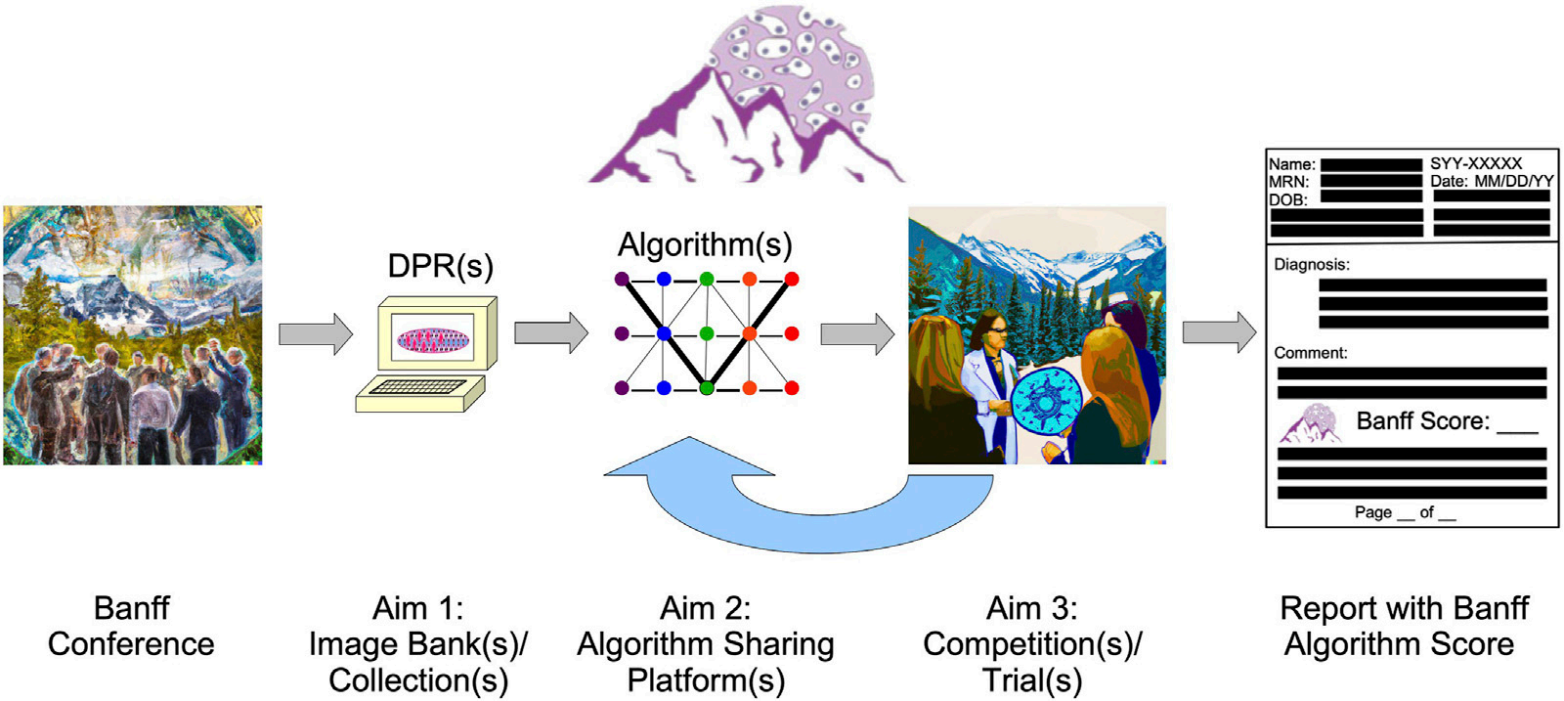
The Banff Digital Pathology Working Group (DPWG) main aims are shown. The primary aims of the Banff DPWG include 1) image bank/collection establishment, to possibly include other data in digital pathology repositories (digital pathology repositories); 2) algorithm sharing platform initialization; and 3) competition/trial organization. Multiple solutions for each of these aims may be possible. After competition/trial conduction among the Banff community and other collaborators, the algorithm performance will be characterized in a process that will affect the future performance and sharing of algorithms; and thus, the competition(s)/trial(s) will provide “feedback” to algorithm sharing. Ultimately, effective, precision patient care could be provided with Banff algorithm scores. (The “Banff Conference” and “Aim 3” image were produced by Kim Solez using DALL-E 2.).

As also detailed in the last Banff Meeting Report [6] and the DPWGs’ first paper [1], the DPWG’s goals are detailed in Tables 1, 2; Figure 1. Notably, future plans can be summarized in three aims:

• Aim 1: Image banks and/or digital pathology repositories for benchmarking algorithms so that research groups can test their AI and other algorithms similar to what is being done in the computer science community overall, with ImageNet supervised natural image classification being a main example.^1^
• Aim 2: Algorithms will developed for the transplant community. One future goal potentially includes the release of “official” Banff algorithms that could be used by the Banff community and beyond. As mentioned in the previous Banff DPWG working group paper, these could include targeted, handcrafted algorithms (e.g., for parameters such as fibrosis, inflammation, steatosis, etc.) [1]; or these could include thoroughly validated AI/ML algorithms. Furthermore, data pipelines for the integration of “–omic” data could be provided so that centers could have mechanisms for mining data within their center as well as sharing with other centers.
• Aim 3: Competitions or trials will be conducted so that groups can compare their algorithms on standard transplant pathology image sets.

**TABLE 1:** Banff digital pathology working group (DPWG) issues and plans. The Banff DPWG issues and future plans are depicted as laid forth in the original DPWG paper (1).

Topic	Items
Issues to address	• Digital automation of pathology practice
	o Computing, Artificial intelligence (AI), Nanotechnology, Machine learning, Slide numeration
Future plans	• Standardization of practices
	• Classification for studies using integrative approaches
	• Interstitial fibrosis and tubular atrophy (IFTA) scoring
	• Inflammation scoring
	• Algorithms to fit to the classification and decrease interobserver variability (e.g., “official” Banff algorithms)
	• Validation of algorithms using slides prepared at different institutions with different laboratory protocols (processing, staining, etc.)
	• Archetypes to be validated across multiple institutions
	• Delivery of precision diagnostic, molecular pathways, and therapeutics (e.g., through established data pipelines and natural language processing)
	• Image bank for groups to test AI and other algorithms

**TABLE 2:** Banff digital pathology working group (DPWG) issues and plans. The Banff DPWG issues and future plans are depicted as further refined through DPWG discussions (1).

Primary goals
Aim 1: Image bank for AI/ML & other algorithms
Aim 2: Algorithms
Algorithm validation using different institutions and laboratory protocols
Algorithms for classification (e.g., “official” Banff)
Banff Parameter algorithms (e.g., IFTA & Inflammation)
Aim 3: Competition/trial
Competition/trial to test algorithms
**Secondary Goals**
Computing, AI/ML, nanotechnology, slide numerationetc.
Standardization of practices
Decrease interobserver variability
Classification using integrative approaches
Precision diagnostics, molecular, & therapeutics, NLP, etc.
Archetypes validated across multiple institutions

Abbreviations: AI/ML, artificial intelligence; machine learning; IFTA, interstitial fibrosis and tubular atrophy; NLP, natural language processing.

This current DPWG paper serves as an update on the DPWG’s progress with selected examples and is not a comprehensive review, and we apologize for related studies that are not cited. The DPWG’s survey research on the current state of digital transplant pathology will be covered, and additional details regarding each of the three aims above will be discussed.

## Image Bank Survey

A survey was conducted from 27 April, 2020, to 23 July, 2020, primarily to determine image bank possibilities for the DPWG. Questions were sent via SurveyMonkey (Palo Alto, California, United States) to both the NEPHROL and NEPHNPPT Discussion Groups (with 701 members and 456 members, respectively) moderated by Kim Solez aimed primarily toward renal pathologists and clinicians interested in renal pathology. The NEPHROL group includes mostly nephrologists and pathologists, and the NEPHNPPT group is a subset of the Renal Pathology Society (RPS) membership.

The Banff DPWG Image Bank Survey had 35 respondents from 13 countries, 19 from the US, 4 Canada, 2 Netherlands, and one each from 10 countries (Supplementary Material). Most (24 or 69% of respondents) specified pathology as their specialty. Of these, 16 (46%) specified that they had a server to manage whole slide images (WSIs) from multiple institutions, and these used various server software and image formats and had a range of storage and bandwidths. In this regard, it is recognized that setting up servers and workflows is quite a complex endeavor; and our survey reflected these complexities [7–10].

Of 13 answering a question regarding the ability of their server to de-identify slide information (including the slide label) automatically, 9 (69%) responded yes; 2 (15%) no; and 2 (15%) not sure. Of 12 answering a question regarding their server’s ability to allow customized and commercial algorithms installation, 8 (67%) answered yes; 2 (17%) no; 1 (8%) only customized algorithms; and 1 (8%) not sure. Of 10 answering a question regarding their server’s ability to allow the correction/standardization of staining variability and other variables in images from multiple laboratories, 9 (90%) answered yes.

Survey questions regarding the possibility of image bank hosting were asked; and of nine responding, 7 (78%) had an associated cost; and only 2 (22%) had no associated cost. The two responding there would be no cost were contacted; and it became clear that one of these would not be able to host the image bank due to logistical issues. Thus, based on the survey, only one respondent at Georgia State University could host an initial image bank pilot. Later discussions in the community revealed another image bank could possibly be hosted at RWTH Aachen University in the future, particularly regarding specimens in Europe subject to European Union General Data Protection Regulation (EU GDPR).

Survey questions also covered existing image bank material available among respondents. Of 28 respondents responding to the question of whether they had an existing transplant WSI repository, 16 (57%) said they had such a repository. When asked for the number of their specimens, the combined specimens included 12,870 kidney, 670 heart, 55 pancreas/islet, 50 lung, 30 liver, 20 intestine, and 2 vascularized composite allograft. Thus, the survey showed that the community already has a substantial specimen number; however, the number of specimens obtained is likely an underestimate.

It is likely that this survey could be repeated in the future with an increased response rate, since interests in AI, ML, and deep learning (DL) are likely increasing [11]. Furthermore, the survey was conducted during the COVID-19 pandemic, which could have hindered response rates. In the future, such a survey could likely find additional servers and material for collaboration.

## Aim 1: Image Bank and Digital Pathology Repository Pilot

Our Banff DPWG conducts discussions, planning, testing, and implementations of appropriate vehicles for pathology AI method dissemination, deployment, and comparison readily accessible by end users. An image bank or digital pathology repository is a goal that the Banff DPWG would like to achieve, similar to the “Big Picture” European digital pathology project,^2^ the Nephrotic Syndrome Study Network (NEPTUNE),^3^ and Kidney Precision Medicine Project (KPMP^4^). In contrast to desktop applications, web-based platforms are preferred by many since they do not require any user-involved installation process [12]. Although some web-based tools have been developed, they are either commercial software with license purchase requirement [12] or limited for new algorithm integration (e.g., Omero [13]).

**FIGURE 2:**
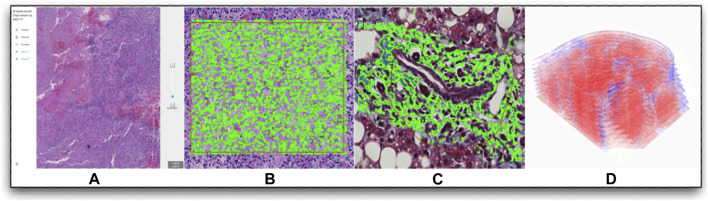
Digital Pathology Laboratory: a publicly available web platform for multi-dimensional pathology image analytics example image manipulations are shown, including the following: **(A)** A representative WSI visualized from DPLab at multiple image resolutions; **(B)** Cell detection result in a user-annotated rectangle region; **(C)** Liver fibrosis quantification with a region annotated by a free-hand annotation tool; **(D)** Detailed 3D liver tissue sub-volume visualization after serial WSI registration and collagen quantifications.

The one respondent available to host a pilot for the DPWG is the Digital Pathology Laboratory (DPLab^5^), a publicly available web platform allowing researchers to visualize, annotate, analyze, and share 2D and 3D pathology images via web-enabled devices. This platform allows users to upload their own WSIs, annotate regions of interest, invoke AI analysis methods, visualize analysis outputs, and download outputs for follow-up statistical comparisons. Due to its web-based framework, DPLab enables WSI annotation and analysis data sharing. Since AI method training and execution relies on a reliable and powerful computational infrastructure, DPLab allows running these AI methods without local computational resources. All requested analysis jobs from the front end are executed through a backend computational environment, addressing a frequent WSI analysis computational obstacle. Currently, DPLab is equipped with numerous WSI analysis algorithms, ranging from color deconvolution, cell detection, nuclei segmentation, histology component quantification, to serial WSI image registration (with some demonstrated in Figure 2). Because DPLab is designed as an open environment, AI methods from the research community can be contributed for method enrichment, validation, and comparison. In the future, additional components are planned for DPLab, such as a quality control component (e.g., similar to those seen in the open-source tool HistoQC [14]). As this software becomes more mature, we envision it and others like it can become useful tools for digital pathology community [12].

Complete digital pathology implementation will require digitization of all workflow steps. For example, in renal pathology, this will require light, immunofluorescence, and electron microscopy digitization. Regarding this, immunofluorescence staining is an integral part of kidney transplant biopsy evaluation, both for C4d staining for detection of antibody-mediated rejection and for immunoglobulins and other complement components for recurrent and *de novo* glomerulonephritis detection. Factors to consider include the ability to support automated scanning with minimal operator input, available immunofluorescence filters, scanning speed, automated tissue detection, image quality, tissue focusing ability, scanning magnification, degree of image bleaching (fading), and price. Major challenges with currently available immunofluorescence slide scanners include inability of scanners to focus on tissue, inability to reflect negative/dim staining, and excessive human technologist time for scanning setup (Dr. Lynn Cornell in DPWG communications).

Digital pathology repositories can include a variety of “omic” data types in the future. Digital pathology “pathomic” data can be included with other “omic” data including genomic, transcriptomic, proteomic, and metabolomic data. “Pathomics” refers to the morphological examination of tissue on the macroscopic, microscopic, and ultrastructural level. “Pathomics” was used at least as far back as a 2007 editorial by Robert Colvin (11, 12) commenting on a study investigating microarray analysis of rejection that later become available in the molecular microscope diagnostic (MMDx) system (13). Using this terminology, the “pathome” can refer to the entirety of morphological histology features, particularly when examined using enhanced ancillary techniques; and enhanced techniques to examine the “pathome” can be termed “Next-Generation histoMorphometry (NGM).” Of note, standard “omic” technologies are increasingly being applied in a “spatial” manner (e.g., spatial transcriptomics and spatial proteomics) [15]. Digital pathology repositories will likely be crucial for the integration of “pathomic” with other “omic” data.

## Aim 2: AI/Deep Learning Algorithms

To effectively develop deep learning (DL)-based support systems for diagnosis and research, including in transplant pathology, three main prerequisites are needed (e.g., when thinking of setting up transplant digital pathology central resources), including: 1) hardware and software infrastructure, 2) interdisciplinary expert teams, and 3) diverse and clinically annotated datasets [16].

(1) The hardware and software infrastructure are becoming more available and affordable, and many pathology labs now have at least partial digital infrastructure. Based on a particular study’s extent and the computational demands of newer DL architectures, however, the introduction of robust digital pathology resources within a single institution can be challenging. Digital pathology and WSIs produce the largest imaging data in clinical medicine. When setting up large digital pathology repositories, sufficient storage capacity is required, which can easily be in the petabyte (PB) range. Such storage must be secure, both in terms of security of access and sufficient backup. Modern DL systems are increasingly computationally expensive to train due to the model size, with many trainable parameters and large datasets. Thus, central high-performance computing (HPC) centers or cloud providers might be needed for model development. Frequently, such HPC centers (or cloud providers) are not used to handling sensitive medical data and privacy concerns (e.g., HIPPA and GDPR); and the legal aspects can be complicated. Also, such centralized algorithmic training requires secure data transfer between institutions. This may also be challenging for security and compliance. Cloud providers and download possibilities can tackle some of these issues. Another potential solution for this could be the use of federated learning approaches, which are becoming more popular not only in computational pathology. These approaches train (parts of the) models on locally stored data (i.e., without the need to move the data from the hospitals) [17–19]. Such federated approaches require scaling up local computing power, which, in our recent experience, is not available everywhere, and sometimes not even considered in some larger repositories. This is not completely surprising, since digital pathology possibilities are still new and emerging. Digital pathology infrastructure maintenance costs (e.g., security updates and other services) need to be kept in mind and can present a challenge when aiming for a long-term digital pathology repository. Thereby, solutions for long-term infrastructure financing are required, and might be a challenge.
(2) AI/DL development and infrastructure maintenance requires experts from information technology (IT), computer science, medicine, research, and other areas [20]. Such an interdisciplinary team is required 1) to ensure a relevant use case and the datasets are defined for meaningful application scenarios in a realistic workflow, 2) a suitable model architecture can be modified to fit the use case, 3) software best practices are followed during training, and 4) to ensure model safety. Ultimately, models should be thoroughly audited before clinical testing, uncovering potential risks and developing mitigation strategies [21]. User studies should test whether systems will be useful in later day-to-day work. The workforce needs of industry vs. academia may be in competition. Generating an environment that motivates IT and AI experts to join academia will be imperative to building up domain-specific expert teams. Also, such teams should have a minimal “critical” size of the particular specialty (e.g., Having only a single AI or IT expert makes the team heavily dependent on a single person, while it does not provide a suitable environment for discussion and exchange for the expert.). It is our experience that large and strongly interdisciplinary teams, directly embedded within the specific application domain might be most efficient in new approach development testing. This direction also helps educate “hybrid” experts (e.g., pathologists with expertise in AI development and AI developers knowledgeable of real-world pathology workflows). Such “hybrid” experts can be augmented by automated systems such as those that help codify the complexity of the Banff classification system [22].
(3) Finally, and currently one of the major challenges in this field, is the availability of relevant, sufficiently large datasets. Sample size is determined by the ML system’s efficiency and the problem complexity. Datasets should be multicentric and reflect the population(s) in which the system will ultimately be used. In addition, it is important to invest time uncovering existing dataset biases before fitting a model to the data and reducing biases as much as possible [23, 24]. To uncover such biases, datasets must be deeply phenotyped, and in the case of pathology, enriched at least with clinical and pathological data. It is essential to validate any DL models using independent cohort(s), which were not used for DL training. While tremendous thought has previously been given to the collection of training datasets [2], only recently have recommendations for the collection of test datasets been issued for the case of computational pathology [20]. Test datasets must be independent from the development datasets. The ML community has long recognized the need for diverse multi-center datasets to reliably assess the generalizability of DL systems. This is now also well established in computational pathology and should be a common standard [2].

One example of how the integration of all prerequisites and joined international cooperation can lead to promising DL algorithm development was previously shown in the DEEPGRAFT study, which involves transplant biopsy weakly-supervised slide-level diagnosis classification using DL [25]. This is currently the largest multicentric dataset of renal transplant biopsies assembled and analyzed centrally, with more than 5,000 WSIs, including consecutive biopsies from a center not included in training, representing a “real-world” scenario and enabling validation and assessment of the model’s generalizability.

Other novel algorithms for efficiently analyzing very large renal tissue biopsy digital WSIs have been integrated into ML pipelines for nephropathology. The developed tools employ a human-AI-loop (HAIL) approach [26] via integrating human with AI for efficiently detecting and segmenting multi-compartmental structures (e.g., glomeruli, tubules, interstitium, and arteries and arterioles). The tool’s performance is shown in computational histologic classification of diabetic nephropathy [27], as well as computational detection and segmentation of interstitial fibrosis and tubular atrophy [28]. The tool has been extended to computationally detect and count podocytes from WSIs, and also subsequent feature extraction for various inference studies [29]. HAIL’s utility has been further shown via integrating the tool with the VIPR (Validated Identification of Pre-Qualified Regions) algorithm [30]. HAIL operates at segmenting large renal structural levels, and VIPR operates at deriving renal micro-compartments using pixel level vector features. In tandem, these tools are being used to conduct unsupervised classification of tubules in the KPMP. Features quantified from HAIL-derived image structures are currently being used for fusing with tissue molecular signatures, such as those derived by CODEX and spatial transcriptomics, to discover new molecularly distinct structural motifs with implications in chronic kidney disease and acute kidney injury. It is anticipated that the tools developed herein will contribute to renal transplant biopsy assessment to automate Banff scoring for chronicity assessment as well as automatically predict graft outcome from pixel level image features.

While retrospective studies have inherent value in showing system applicability or useability, prospective evidence of the clinical benefit of DL systems must be generated through well-designed clinical trials. Promising studies include those examining the classification of rejection versus other diseases [25] and antibody-mediated rejection under Banff criteria [31] in the kidney; and in cardiac endomyocardial biopsies, allograft rejection has been distinguished from benign mimics (Quilty B lesions) using AI [32]. However, clinical trials implementing DL systems are currently largely missing in the field of computational pathology, but in some scenarios might also be hard to provide.

## Aim 3: Competition/Trial and Current Image Analysis Trial Work

**FIGURE 3:**
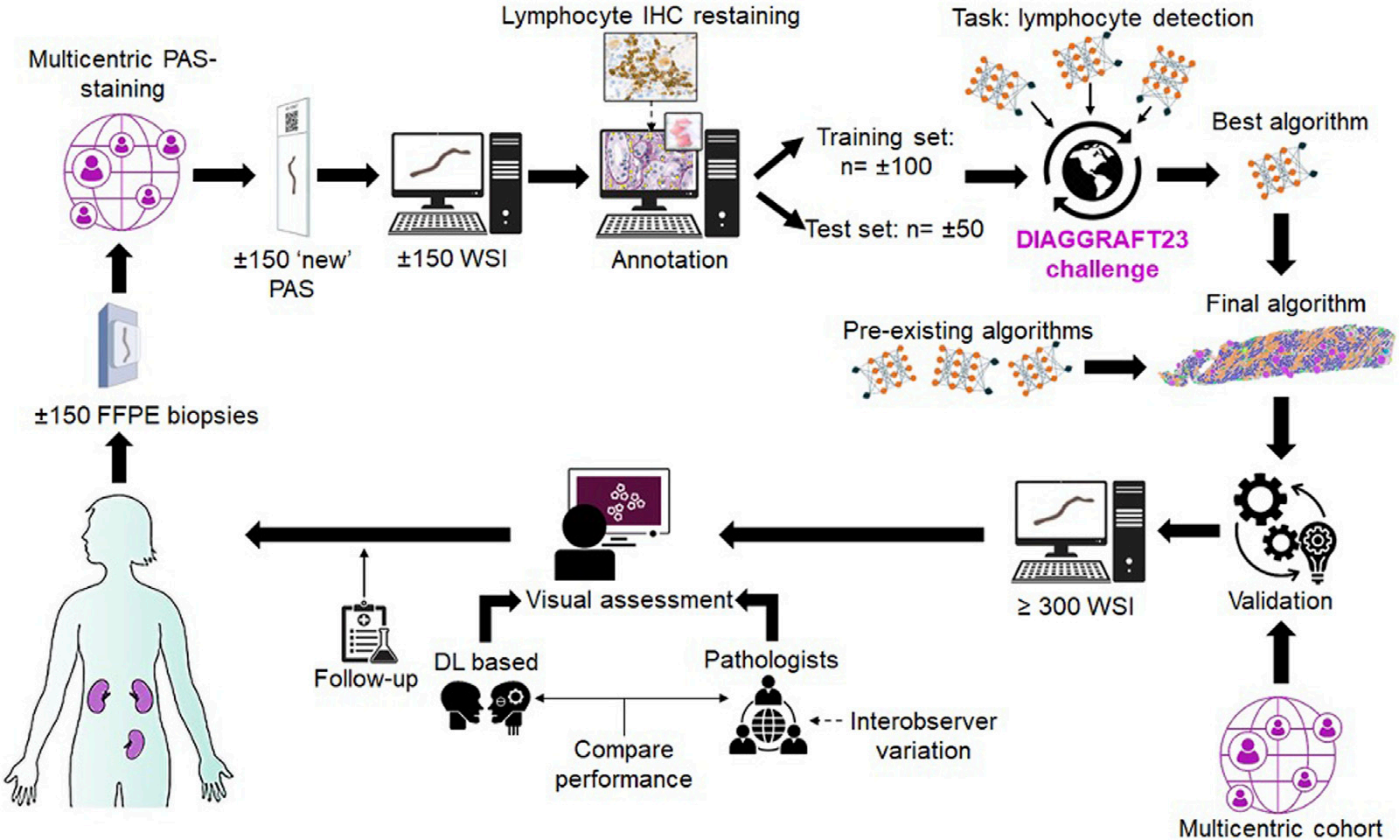
The DIAGGRAFT Challenge Work Plan is shown. Abbreviations: FFPE, formalin-fixed paraffin-embedded; PAS, periodic acid Schiff; WSI, Whole Slide Images; IHC, immunohistochemistry.

As mentioned previously, our last aim deals with competitions or trials will be conducted so that groups can compare their algorithms on standard transplant pathology image sets. In this regard, the Banff DPWG has an ongoing collaboration that has been discussed in DPWG meetings entitled “DIAGGRAFT: leveraging artificial intelligence technology for accurate quantitative histological diagnostic assessment of transplant renal biopsies.” The Dutch Kidney Foundation recently awarded a Success Accelerator Grant for the DIAGGRAFT project. DIAGGRAFT was started in January 2022 by Dominique van Midden, Meyke Hermsen, Jeroen van der Laak, et al, and will be executed in close collaboration with the DPWG. This project builds upon former research by Hermsen et al. [33, 34] that developed AI (more specifically: DL) for automated assessment of histopathologic features in digitized kidney tissue sections. DIAGGRAFT aims to take developed AI a step further, extending these techniques and preparing them for large-scale research- and even diagnostic use. The DIAGGRAFT consortium will organize a so-called “grand challenge”: an international competition, similar to challenges previously organized (e.g., PANDA [35] for prostate cancer,^6^ CAMELYON [36–38] for breast cancer and lymph node metastasis, and other Kaggle efforts^7^ ^,^ ^8^). In the DIAGGRAFT challenge, a large, annotated, multi-center data set will be established and provided to participants with the goal to collectively build AI for inflammatory cell detection in periodic-acid Schiff-stained slides. The best inflammatory cell detection model(s) from the DIAGGRAFT challenge will be combined with existing structure segmentation AI to quantify Banff classes. In the last part of DIAGGRAFT, AI will be validated on a large patient cohort, originating from multiple international medical centers and scored by an expert renal pathologist panel. DIAGGRAFT aims to develop powerful DL tools for objective and reproducible Banff scoring, validated in a multicenter setting against graft function and survival. The resulting DL models will be made available to the Banff community for subsequent validation studies. DIAGGRAFT is visually displayed in Figure 3.

## Conclusion

The Banff DPWG plans to continue the efforts of fostering the establishment of image banks and digital pathology repositories, of stimulating algorithm development, and supporting the validation of these algorithms. The DPWG’s efforts will be disseminated through a variety of venues (e.g., during the annual meeting of the American Society of Transplantation), to stimulate engagement of the entire transplant community. Funding sources are being explored to financially support efforts of the DPWG. Digital pathology techniques allow computational pathology, which provides automated histopathology analyses with more throughput scalability, reproducibility, and precision [5, 15, 39–42]. Indeed, these new technologies will essentially allow numerous novel manipulations, such as the translation/augmentation of one stain to another [43, 44]. It is possible that AI/ML will serve as a “gold standard” in some sense, although we foresee AI/ML augmenting pathologists rather than replacing them as the “gold standard.” Algorithms and other advances for the Banff community may eventually arise from these efforts, with the ultimate goal of providing more effective, precision patient care.

## Footnotes

1
https://www.image-net.org/

2
https://bigpicture.eu/

3
https://www.neptune-study.org/

4
https://www.kpmp.org

5
https://dplab.gsu.edu/

6
https://www.kaggle.com/c/prostate-cancer-grade-assessment/overview

7
https://www.kaggle.com/c/hubmap-kidney-segmentation, https://cns.iu.edu/docs/publications/2021-Godwin-FTUs.pdf

8
https://hubmapconsortium.github.io/ccf/pages/kaggle2.html

## Abbreviations

2D, 3D, 2-dimensional, 3-dimensional; AI, Artificial intelligence; DL, deep learning; DPWG, digital pathology working group; EU GDPR, European Union General Data Protection Regulation; HPC, High-performance computing; IFTA, interstitial fibrosis and tubular atrophy; IT, information technology; ML, machine learning; NLP, natural language processing; WSI, whole slide image/imaging.
