# Supplementary material for: Banff Digital Pathology Working Group: Image Bank, Artificial Intelligence Algorithm, and Challenge Trial Developments
Source: Transpl Int. 2023 Oct 16;36:11783. doi: 10.3389/ti.2023.11783 (PMC10614670; doi:10.3389/ti.2023.11783)
Supplement: Supplementary file 1 [file DataSheet1.pdf]

Banff Digital Pathology Working Group (DPWG):
Image Bank, Artificial Intelligence Algorithm, and Challenge Trial Developments

SUPPLEMENT

BANFF DIGITAL PATHOLOGY WORKING GROUP (DPWG): IMAGE BANK, ARTIFICIAL INTELLIGENCE ALGORITHM, AND CHALLENGE TRIAL DEVELOPMENTS 1

SUPPLEMENT 1

SURVEY QUESTIONS AND RESPONSE NOTES 3

Question 1: Please specify the following: 3

Question 2: Do you have a computerized server that can manage whole slide images (WSIs) from multiple institutions? 4

Question 3: What software is used on the server? 4

Question 4: What WSI image formats are supported? 4

Question 5: Can the server application de-identify slide information (including the slide label) part automatically? 4

Question 6: Does the server allow the installation of customized and commercial algorithms: 5

Question 7: Does your software allow the correction/standardization of staining variability and other variables in the images from multiple laboratories? 5

Question 8: Please provide exact amount of storage available: (in gigabytes[GB], terabytes [TB], petabytes[PB], etc.) OR please provide a comment (unknown, to be determined,etc.] 5

Question 9: Please provide the bandwidth of the internet connection: (in megabytes [GB]/second [s], gigabytes [GB]/s, terabytes [TB]/s,petabytes[PB]/s, etc.) OR please provide a comment (unknown, to be determined, etc.] 5

Question 10: Will there be a cost associated with hosting the image bank? 6

Question 11: Please specify the amount required in United States $: 6

Question 12: Do you have an existing whole slide image (WSI) repository of transplant specimens? 6

Question 13: How many specimens (e.g., biopsies, explants, etc.) are in your repository for the following organ systems? (If you have none, please put “0”.) 7

Question 14: What is the WSI digital file format of your images? 7

Question 15: If you would like, please provide additional comments on your existing repository: 7

Question 16: Please provide an overall comment (optional, if you desire): 8

Survey Questions and Response Notes

Question 1: Please specify the following:

Name
E-mail address
Institution
Department
Country

The Banff Digital Pathology Working Group (DPWG) Image Bank Survey Countries included the following:

Country	# of Respondents	% of Respondents
United States of America	19	54%
Canada	4	11%
Netherlands	2	6%
Belgium	1	3%
Brazil	1	3%
China	1	3%
Columbia	1	3%
Costa Rica	1	3%
Egypt	1	3%
Germany	1	3%
Hungary	1	3%
India	1	3%
Nepal	1	3%
	35 respondents total	

The Banff Digital Pathology Working Group (DPWG) Image Bank Survey Department/Specialties included the following:

Specialty/Department	# of Respondents	% of Respondents
Pathology	24	69%
Nephrology	3	9%
Mathematics and statistics	1	3%
Neurology	1	3%
“Transplantation division”	1	3%
No response available	5	14%
	35 respondents total	

Question 2: Do you have a computerized server that can manage whole slide images (WSIs) from multiple institutions?

	# of Respondents	% of Respondents
Yes	16	46%
No	19	54%
	35	

Question 3: What software is used on the server?

(e.g., Leica/Aperio, Philips, DigitalSlide Archive, PathPresenter, Sectra, Customized/Developed at our Institution, etc.)

> Software present on the servers included Leica/Aperio, Philips, PathPresenter, Sukraa 3DHistech, Apollo AARC, Digital Slide Archive and other customized programs. WSI slide formats used included Leica/Aperio svs, Hamamatsu ndpi, TIFF, 3DHistech, and other customized formats.

Question 4: What WSI image formats are supported?

(e.g., Leica/Aperio svs, Olympus vsi, Hamamatsu ndpi, tiff, 3DHISTECH, etc.)

> The following were listed: 3Dhistech, jp2, Leica/Aperio svs, Hamamatsu ndpi, TIFF.
> One specified, "All formats supported by https://openslide.org/, also MRXS".

Question 5: Can the server application de-identify slide information (including the slide label) part automatically?

Yes/No

If yes, could you please comment on the process:

> Of 13 respondents answering this question, 9 (69%) responded yes; 2 (15%) no; and 2 (15%) not sure.

	# of Respondents	% of Respondents
Yes	9	69%
No	2	15%
Not sure	2	15%
	13	

Question 6: Does the server allow the installation of customized and commercial algorithms:

Yes/No.
If yes, could you please comment on the process for this installation:

Of 12 respondents answering this question, 8 (67%) answered yes; 2 (17%) no; 1 (8%) only customized algorithms; and 1 (8%) not sure.

	# of Respondents	% of Respondents
Yes	8	67%
No	2	17%
Only customized algorithms	1	8%
Not sure	1	8%
	12	

Question 7: Does your software allow the correction/standardization of staining variability and other variables in the images from multiple laboratories?

Yes/No
If yes, could you please comment on this:

Of 10 respondents answering this question, 9 (90%) answered yes.

	# of Respondents	% of Respondents
Yes	9	90%
No	1	10%
	10	

Question 8: Please provide exact amount of storage available: (in gigabytes[GB], terabytes [TB], petabytes[PB], etc.) OR please provide a comment (unknown, to be determined,etc.]

Specific answers were received from 3 respondents, ranging from 8 to 160 TB; and 6 responded that this was unknown or to be determined.

Question 9: Please provide the bandwidth of the internet connection: (in megabytes [GB]/second [s], gigabytes [GB]/s, terabytes [TB]/s,petabytes[PB]/s, etc.) OR please provide a comment (unknown, to be determined, etc.]

There were 10 respondents who answered this question. Specific answers were received from 4 (40%) respondents, ranging from 100 Mbit/second to 10 GB/second; and 6 (60%) responded that this was unknown or to be determined.

Question 10: Will there be a cost associated with hosting the image bank?

Of 9 responding regarding this question, 7 (78%) responded that there would be a cost associated with this; and only 2 (22%) responded that there would be no cost.

	# of Respondents	% of Respondents
Yes	7	78%
No	2	22%
	9	

Question 11: Please specify the amount required in United States $:

Respondents were asked a question on both the initial amount and the maintenance amount:

The initial amount required ranged from unknown or “to be determined” to $25,000.

The maintenance amount ranged from unknown, “to be determined”, or “thousands”, to $20,000.

Question 12: Do you have an existing whole slide image (WSI) repository of transplant specimens?

Of 28 respondents responding to the question of whether they had an existing WSI repository of transplant specimens, 16 (57%) said that they had such a repository; and 12 (43%) said that they do not have such a repository.

	# of Respondents	% of Respondents
Yes	15	57%
No	12	43%
	28	

Question 13: How many specimens (e.g., biopsies, explants, etc.) are in your repository for the following organ systems? (If you have none, please put “0”.)

The combined number of specimens included the following: 12,870 kidney, 670 heart, 55 pancreas/islet, 50 lung, 30 liver, 20 intestine, and 2 vascularized composite allograft.

Organ	# of Specimens
Kidney	12,870
Heart	670
Pancreas/islet	55
Lung	50
Liver	30
Intestine	20
Vascularized composite allograft	2

Question 14: What is the WSI digital file format of your images?

Current image formats utilized for existing image banks at respondent institutions included Aperio svs, iSyntax, iSyntax (Philips) converted to bigTIFF (Philips), jp2 files (that can be opened with Aperio ImageScope), mxrs/MRXS, and ndpi.

Question 15: If you would like, please provide additional comments on your existing repository:

Responses included the following (with some individual identifying information removed):

- “with the onset of COVID we are trying to go digital so that faculty can sign out from home, under somewhat more relaxed guidelines. … Current challenges include finding a dedicated staff to operate and troubleshoot the instrument, appropriate funneling of requests - currently there is no process map for what is scanned when, therefore some delays. Renal biopsy provides additional challenges because of immunofluorescence which is currently not being scanned. File storage required collaboration with IT department to create a special storage drive.”
- “DEEPGRAFT cohort (multicenter, multinational). Index and protocol biopsies from around 2000 patients, H&E, PASD and silver stained slides for each biopsy. Clinical annotation (biopsy diagnosis labels) on around 1000 cases, incl Banff lesion scores by 3 observers”
- “Each pathologist interested saved cases.”
- “Renal allograft biopsies from NIH-funded … study…”

- “We have a mixture of adult & pediatric transplant biopsies.”

Question 16: Please provide an overall comment (optional, if you desire):

Responses included the following (with some individual identifying information removed):

- “… We are currently scanning for real time use. Our only "repositories" are for Education purposes (not tissue or utility dependent) or research (non-clinical)…”
- “We … have a workload of close to 800 renal allograft biopsies per year which can be used for the study.”
- “Although we cannot allow users from other institutions. The images could be shared for teaching purposes.”
- “The repository has been collected by me, but I do not have ownership of it; it belongs to the NIH-funded … consortium.”
